# Interactions between BMP-7 and USAG-1 (Uterine Sensitization-Associated Gene-1) Regulate Supernumerary Organ Formations

**DOI:** 10.1371/journal.pone.0096938

**Published:** 2014-05-09

**Authors:** Honoka Kiso, Katsu Takahashi, Kazuyuki Saito, Yumiko Togo, Hiroko Tsukamoto, Boyen Huang, Manabu Sugai, Akira Shimizu, Yasuhiko Tabata, Aris N. Economides, Harold C. Slavkin, Kazuhisa Bessho

**Affiliations:** 1 Department of Oral and Maxillofacial Surgery, Graduate School of Medicine, Kyoto University, Shogoin-Kawahara-cho, Sakyo-ku, Kyoto, Japan; 2 Department of Paediatric Dentistry, School of Medicine and Dentistry, James Cook University, Cairns, Australia; 3 Translational Research Center, Kyoto University Hospital, Shogoin-Kawahara-cho, Sakyo-ku, Kyoto, Japan; 4 Laboratory of Host Defense, World Premier International Immunology Frontier Research Center, Osaka University, Suita, Osaka, Japan; 5 Department of Biomaterials, Institute for Frontier Medical Sciences, Kyoto University, Kyoto, Japan; 6 Regeneron Pharmaceuticals, Tarrytown, New York, United States of America; 7 Center for Craniofacial Molecular Biology, Division of Biomedical Sciences, Ostrow School of Dentistry, University of Southern California, Los Angeles, California, United States of America; University of Colorado, Boulder, United States of America

## Abstract

Bone morphogenetic proteins (BMPs) are highly conserved signaling molecules that are part of the transforming growth factor (TGF)-beta superfamily, and function in the patterning and morphogenesis of many organs including development of the dentition. The functions of the BMPs are controlled by certain classes of molecules that are recognized as BMP antagonists that inhibit BMP binding to their cognate receptors. In this study we tested the hypothesis that USAG-1 (uterine sensitization-associated gene-1) suppresses deciduous incisors by inhibition of BMP-7 function. We learned that USAG-1 and BMP-7 were expressed within odontogenic epithelium as well as mesenchyme during the late bud and early cap stages of tooth development. USAG-1 is a BMP antagonist, and also modulates Wnt signaling. USAG-1 abrogation rescued apoptotic elimination of odontogenic mesenchymal cells. BMP signaling in the rudimentary maxillary incisor, assessed by expressions of Msx1 and Dlx2 and the phosphorylation of Smad protein, was significantly enhanced. Using explant culture and subsequent subrenal capsule transplantation of E15 USAG-1 mutant maxillary incisor tooth primordia supplemented with BMP-7 demonstrated in USAG-1^+/−^ as well as USAG-1^−/−^ rescue and supernumerary tooth development. Based upon these results, we conclude that USAG-1 functions as an antagonist of BMP-7 in this model system. These results further suggest that the phenotypes of USAG-1 and BMP-7 mutant mice reported provide opportunities for regenerative medicine and dentistry.

## Introduction

A significant number of discoveries have also been advanced for the establishment of tooth position and patterning, critical developmental pathways that regulate cell and tissue formations, extracellular matrix formations, biomineralization, and the associated genes and gene families (see recent reviews by [1]–[3]).

A curious clinical aberration during craniofacial morphogenesis is the patterning and subsequent organogenesis of supernumerary tooth organs. The term “supernumerary teeth” describes the production of more than the normal number of teeth in the human primary or permanent dentition. The prevalence of supernumerary teeth on a population basis ranges from 0.1 to 3.6% [4], [5]. In contrast, normal mouse development presents a monophyodont dentition composed of one incisor and three molars in each quadrant. Unlike humans, mice have only molar and incisor tooth organs separated by a “toothless region” termed the diastema. In addition, mice have a single primary dentition and their teeth are not replaced.

The animal models have significantly contributed towards understanding the molecular and developmental biology of human craniofacial biology (see treatise by [6]). A number of mouse mutants provide insights into the supernumerary tooth formation [7]–[20]. Several mechanisms by which supernumerary tooth might arise in mice have been proposed [21]–[26]. One plausible explanation for supernumerary tooth formation is the rescue of tooth rudiments such as within the diastema region [26]–[29] or maxillary deciduous incisor [15], [30]. During early stages of mouse tooth development transient vestigial tooth buds develop in the diastema area; developing to the bud stage yet later regressing and disappear by apoptosis, or merge with the mesial crown of the adjacent first molar tooth organ [26], [28], [29]. The rudimentary maxillary incisor regressed by apoptotic elimination of mesenchymal cells [15]. Recently, we demonstrate that USAG-1(also known as Sostdc1, ectodin, and Wise) -deficient mouse model has supernumerary incisors in the maxillary and mandible, a fused tooth in the maxillary and mandibular molar regions, and a supernumerary tooth was also located in front of the first mandibular molar [15]. Increased BMP signaling results in supernumerary teeth in the USAG-1-deficient mouse model [21].

USAG-1 is a bone morphogenetic protein antagonist that is expressed at high levels in the kidney and inhibits BMP-7 bioactivity [31], [32]. Bone morphogenetic protein-7 is a 35-kDa homodimeric protein, and plays an important role in the specification and patterning of the early embryo and functions to regulate apoptosis in many developmental processes [33], [34]. BMP-4 as well as BMP-2 and BMP-7 are expressed in the limb bud [35], and in cranial neural crest cells [36], [37] with associated induction of apoptosis. Curiously, BMP-4 and BMP-7 prevent apoptosis of the metanephric mesenchyme during kidney development [38], [39]. Further, as the result from renal injury, BMP-7 inhibits apoptosis of proximal tubule epithelial cells [40]. It has been reported that USAG-1 binds to BMP-7 and inhibits the apoptosis-protective actions of BMP-7 in the kidney [41]. BMP-7 null mice present a craniofacial syndrome including severe eye defects, including anophthalmia and microphthalmia, skeletal and renal anomalies, and die shortly after birth [38], [42]–[44]. Meanwhile, absence or agenesis of the maxillary teeth in conditional BMP-7 null mice has recently been reported [44].

The purpose of these present investigations is to test the hypothesis that USAG-1 suppresses deciduous incisors by inhibition of BMP-7 function. If valid, our results would also demonstrate that a novel BMP-7 antagonist functioning as a negative regulator in BMP functions can assist towards advancing regenerative medicine and dentistry.

## Materials and Methods

### Ethic Statement

All procedures were approved by the Animal Care Committee at Kyoto University.

### Mouse strains

USAG-1/LacZ mice [45] and BMP-7/LacZ mice [46] were used in this study. USAG-1/LacZ mice were on a C57Bl6/J background and BMP-7/LacZ mice were on an Imprinting Control Region (ICR) background. USAG-1^−/-−^/BMP-7^−/-−^ mice were generated by crossing two lines of mice. To eliminate the influence of mouse background, only F2 progeny was analysed. Embryos were obtained by timed mating, day E0 started from midnight prior to finding a vaginal plug.

### Whole-mount LacZ staining

Embryos thus obtained were briefly washed in Hank's solution and immediately fixed in cold fixative solution [2% formaldehyde, 0.2% glutaraldehyde, 0.01% sodium deoxycholate and 0.02% NP-4 in phosphate buffer saline (PBS)] for 2 min. They were subsequently washed several times with PBS/2 mM MgCl_2_, and stained for several hours to overnight in x-gal staining solution (0.1 M phosphate buffer pH 7.3, 2 mM MgCl_2_, 0.01% sodium deoxycholate, 0.02% NP-40, 5 mM K_3_Fe(CN)_6_, 5 mM K_4_Fe(CN)_6_ and 1 mg/ml x-gal) at room temperature in the dark.

Embryos were then washed in PBS, post-fixed in 1% paraformaldehyde (PFA) and dissected for macroscopic analysis.

### LacZ staining on sections

Embryos obtained from timed mating were fixed in 4% PFA, equilibrated in 25% sucrose and embedded in water soluble glycols and resins (Miles Laboratories, Elkhart, IN). Sections of 8 µm were cut and stained for LacZ following the same protocol as for whole-mount staining except that they were fixed for 5 min and stained at 37°C. Sections were post-fixed in 1% PFA, counter-stained with nuclear fast red, mounted with glycerine, covered and sealed with nail polish.

### Analysis of tooth phenotype

Embryos and neonates were fixed in 4% PFA and embedded in paraffin. Sections of 7 mm were cut and stained with haematoxylin and eosin. The area of the maxillary rudimentary incisor tooth of all mice was measured using Image J software (US NIH, Bethesda, MD, USA).

### Detection of apoptosis

Apoptosis was detected by the terminal deoxynucleotidyl transferase-mediated dUTP nick end-labelling method using an ApopTag Plus In Situ Apoptosis Detection Kit-Fluorescein (Oncor, Rockville, MD) according to the manufacturer's specifications. Specimens were briefly washed, dehydrated through a graded series of ethanol in PBS and subjected to labelling with an ApopTag Plus In Situ Apoptosis Detection Kit. Cell nuclei were counter stained with instant-blue nuclear probe fluorescing (455 nm) compound (SouthernBiotech, Birmingham, AL).

### Immunohistochemistry

Paraffin-embedded sections of embryos were immunostained with primary rabbit polyclonal antibodies against phosphorylated Smad 1/5/8 (1∶100; Cell Signaling Technology, Beverly, MA); goat polyclonal antibodies against phosphorylated Smad 2/3 (1∶100; Santa Cruz Biotechnology Inc., Santa Cruz, CA); and secondary biotinylated anti-rabbit, goat and mouse antibodies (Nichirei Bioscience, Tokyo, Japan), as previously described [41], [47], [48]. Sections were then counter-stained with haematoxylin, dehydrated in a graded series of ethanol and xylene, and covered with coverslips.

### Whole mount *in situ* hybridization

Specific probes for mouse Dlx2 and Msx1 were obtained by the reverse transcription-polymerase chain reaction method and confirmed by direct sequencing. Digoxigenin (DIG)-labelled sense and antisense riboprobes were prepared by the in vitro transcription of phagemids using an RNA Transcription Kit (Stratagene, La Jolla, CA) according to the manufacturer's specifications. Whole mount in situ hybridization was performed according to the following protocol. Briefly, specimens were fixed in 4% PFA in PBS and permeabilized with Radioimmunoprecipitation assay buffer, following which they were hybridized overnight with 1 mg/ml DIG-labelled riboprobes at 70 °C. The specimens were then washed, blocked, and further incubated with alkaline phosphatase-conjugated anti-DIG (Boehringer Mannheim, Indianapolis, IN) at a 1∶2000 dilution at 4°C overnight. The bound alkaline phosphatase was visualized after incubation with nitro blue tetrazolium (NBT)/5-bromo-4-chloro-3-indolyl phosphate (BCIP) substrate.

### Organ culture and subrenal capsule assay

E15 USAG-1-deficient, heterozygous, and wild-type mice incisors were dissected in Hank's solution under a stereomicroscope. Tooth explants were cultured for one day on Nucleopore filters at 37°C in 5% CO_2_ in a Trowell-type organ culture containing BGJb with 10% fetal bovine serum. The explants were then transplanted beneath the kidney capsule. Gelatin hydrogel microspheres (MedGel, Osaka, Japan) with <30 µm diameter were prepared as described previously [49], [50]. The microspheres were incubated with PBS (control) or PBS containing BMP-7 (R&D Systems, Minneapolis, MN; 200 ng/ml) for 1 h at room temperature. Subcutaneous implantation was performed using a pair of fine tweezers under the stereomicroscope. Animals were sacrificed at 19 days after transplantation. Explants were fixed in 10% PFA and processed for immunohistochemistry.

### Statistical analysis

Data were analysed by two-way analysis of variance and Student's t-test, and significance was determined at a confidence level of p<0.01. All experiments were performed in triplicate.

## Results

### BMP-7 co-localization with USAG-1 in mesenchymal and epithelial cells of the maxillary rudimentary incisor tooth germ

**Figure 1:**
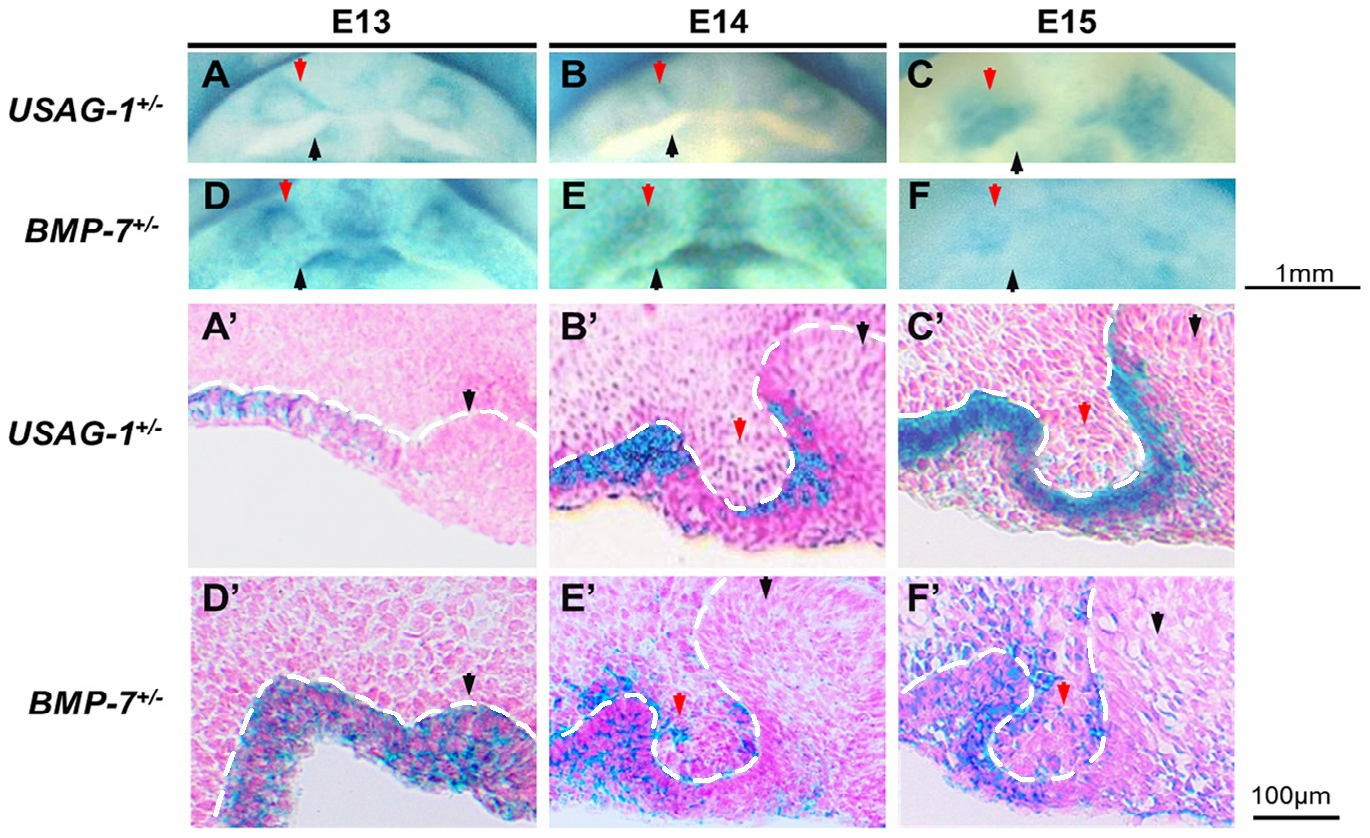
BMP-7 co-localization with USAG-1 in the mesenchymal and epithelial cells of maxillary rudimentary incisor. (A–F) Whole-mount X-Gal expression in tooth germs of E13 –15 maxillary. (A’–F’) Parasagittal sections (anterior to the left) of the tooth germs from panels A–F show X-Gal expression in the rudimentary incisor epithelium. USAG-1 (A–C, A’–C’) and BMP-7 (D–F, D’–F’) were expressed in the tooth organ of rudimentary maxillary incisor (red arrow) in addition to the tooth organ of characteristic incisor (black arrow). At E13, USAG-1 and BMP-7 transcripts were prominent in the labial epithelium in addition to the dental epithelium (A, D, A’ and D’). At E14, USAG-1 and BMP-7 started to be expressed in the mesenchymal cells of the maxillary rudimentary incisor to the surface of the epithelium (B, E, B’ and E’). At E15, the expression of both USAG-1 and BMP-7 increased in the mesenchymal cells of the maxillary rudimentary incisor (C, F, C’ and F’). BMP-7 co-localized with USAG-1 in the area of the tooth germ of maxillary rudimentary incisor in addition to the tooth organ of regular maxillary incisor. White dotted line indicates the interface between epithelium and mesenchyme.

USAG-1 transcript expression was detected in the area of the maxillary rudiment incisor tooth germ in addition to the regular maxillary incisor tooth organ by in situ hybridization [15]. We examined the expression of USAG-1 and BMP-7 in the maxillary rudiment incisor tooth germ at E13–15 using USAG-1 ^+/LacZ^ and BMP-7^+/LacZ^ mice. At E13 (late bud stage), USAG-1 and BMP-7 transcripts were prominent in the labial epithelium in addition to the enamel organ epithelium (Fig. 1A, D, A’ and D). At E14 (early cap stage), USAG-1 and BMP-7 transcripts were first detected in the mesenchymal cells of the maxillary rudimentary incisor (Fig. 1B, E, B’ and E’). At E15 (cap stage), USAG-1 and BMP-7 expression increased in the mesenchymal cells of the maxillary incisor tooth organ (Fig. 1C, F, C’ and F’). BMP-7 co-localized with USAG-1 in the area of the maxillary rudiment incisor tooth organ in addition to the conventional maxillary incisor tooth organ.

### USAG-1 functions as a BMP-7 antagonist in maxillary supernumerary incisor formation

**Figure 2:**
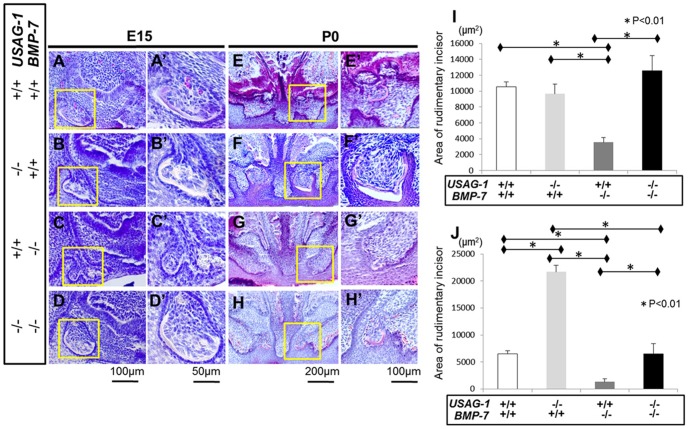
USAG-1 antagonises BMP-7 in maxillary supernumerary incisors formation. Sagittal sections of E15 (A–D) embryos and frontal sections of mice on the day of birth (E–H). (A’–H’) Higher magnification of the boxed regions in (A–H). USAG-1^+/+^/BMP-7^+/+^, (A, A’, E, E’); USAG-1^−/−^/BMP-7^+/+^, (B, B’, F, F’); USAG-1^+/+^/BMP-7^−/−^, (C, C’, G, G’) and USAG-1^−/−^/BMP-7^−/−^ (D, D’, H, H’). The area of rudimentary incisor was measured in transverse sections of USAG-1^+/+^/BMP-7^+/+^ (white bars), USAG-1^−/−^/BMP-7^+/+^ (right grey bars), USAG-1^+/+^/BMP-7^−/−^ (dark grey bars) and USAG-1^−/−^/BMP-7^−/−^ (black bars) mice (n = 5) in E15 (I) and P0 (J). At E15, the area of the maxillary deciduous incisor was identified in wild type as well as all mutant mice in the labial border of the epithelial invagination. The size of rudimentary incisor is similar except USAG-1^+/+^/BMP-7^−/−^ at E15 (A, A’, B, B’, C, C’, D, D’ and I).Rudimentary tooth primordia in USAG-1^−/−^/BMP-7^−/−^ and USAG-1^+/+^/BMP-7^+/+^ regressed and its size became smaller at birth, whereas the teeth in USAG-1^−/−^/BMP-7^+/+^ continued to develop and enamel organ was formed (E, E’, F, F’, H, H’ and J).

**Figure 3:**
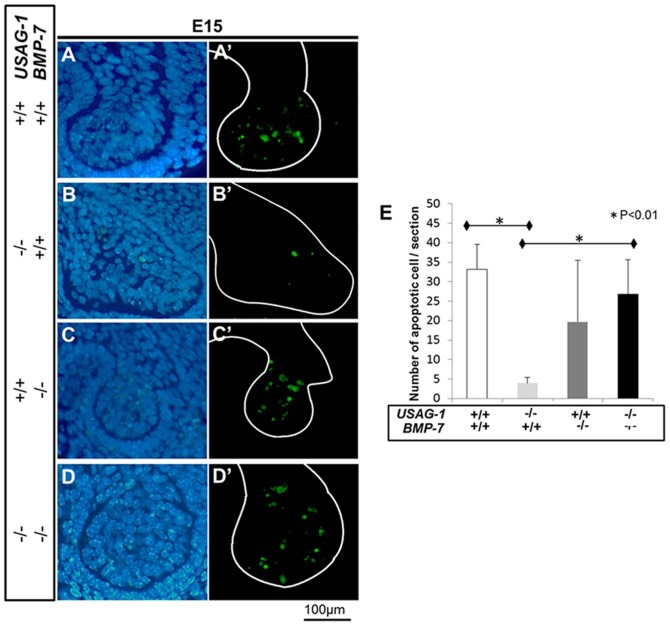
USAG-1 abrogation rescues apoptotic elimination of odontogenic mesenchymal cells. Sagittal sections of E15 embryo maxillary rudimentary incisor in transferase-mediated dUTP nick end-labelling method (TUNEL) staining; Cell nuclei were counterstained with Dapi (A–D), and TUNEL-positive cells in mesenchymal cells of maxillary rudimentary incisor (A’–D’). USAG-1^+/+^/BMP-7^+/+^, (A, A’); USAG-1^−/−^/BMP-7^+/+^, (B, B’); USAG-1^+/+^/BMP-7^−/−^, (C, C’) and USAG-1^−/−^/BMP-7^−/−^ (D, D’). White line indicates the interface between epithelium and mesenchyme. The number of TUNEL-positive cells per section was counted in transverse section of USAG-1^+/+^/BMP-7^+/+^ (white bars), USAG-1^−/−^/BMP-7^+/+^ (right grey bars), USAG-1^+/+^/BMP-7^−/−^ (dark grey bars), and USAG-1^−/−^/BMP-7^−/−^ (black bars) mice (n = 3; E). USAG-1 abrogation rescued the apoptotic elimination of odontogenic mesenchymal cells in the tooth primordia of rudimentary maxillary incisor at E15, whereas these size are comparable (A, A’, B and B’). The apoptotic odontogenic mesenchymal cells in USAG-1^−/−^/BMP-7^−/−^ are similar to USAG-1^+/+^/BMP-7^+/+^ in contrast to those in USAG-1^−/−^/BMP-7^+/+^ (A, A’, B, B’, D and D’).

BMP-7 deficient mice die shortly after birth due to severe renal hypoplasia [38], [42]. To test the hypothesis that USAG-1 functions as a novel BMP-7 antagonist in maxillary supernumerary incisors formation, we analysed adult USAG-1^−/−^/BMP-7^+/−^ mice. The incidence or pattern of supernumerary incisors formation in USAG-1^−/−^/BMP-7^+/+^ and USAG-1^−/−^/BMP-7^+/−^ mice are almost identical, which was about 50% (Table S1). We previously demonstrated that the supernumerary maxillary incisor formed as a result of the successive development of the rudimentary incisor tooth primordia [15]. Therefore, we analysed the maxillary rudiment incisor tooth germ of USAG-1^−/−^/BMP-7^−/−^mice in select embryonic stages. We performed a series of histological investigations of USAG-1^+/+^/BMP-7^+/+^, USAG-1^−/−^/BMP-7^+/+^, USAG-1^+/+^/BMP-7^−/−^ and USAG-1^−/−^/BMP-7^−/−^ mice at E15 and newborn (P0). At E15, the area of the maxillary deciduous incisor was identified in wild type as well as all mutant mice in the labial border of the epithelial invagination (as described by [15], [51]). The size of rudimentary incisor is similar except USAG-1^+/+^/BMP-7^−/−^ at E15 (Fig. 2A, A’, B, B’, C, C’, D, D’ and I). Rudimentary tooth primordia in USAG-1^−/−^/BMP-7^−/−^ regressed and their size regressed and became smaller at birth. This was also observed for USAG-1^+/+^/BMP-7^+/+^ whereas the tooth organs in USAG-1^−/−^/BMP-7^+/+^ continued to develop and the enamel organ was formed (Fig. 2, E, E’, F, F’, H, H’ and J). USAG-1 abrogation rescued the apoptotic elimination of odontogenic mesenchymal cells in the rudimentary maxillary incisor tooth primordia at E15, whereas the size remained comparable (Fig. 3 A, A’, B and B’) [15]. The apoptotic mesenchymal cells in USAG-1^−/−^/BMP-7^−/−^are similar to USAG-1^+/+^/BMP-7^+/+^ in contrast to that of USAG-1^−/−^/BMP-7^+/+^ (Fig 3.A, A’, B, B’, D and D’). These results demonstrate that USAG-1 functions as a BMP-7 antagonist in maxillary supernumerary incisors formation.

### Increased BMP signaling in supernumerary teeth of the USAG-1 deficient mice is prohibited by BMP-7 abrogation

**Figure 4:**
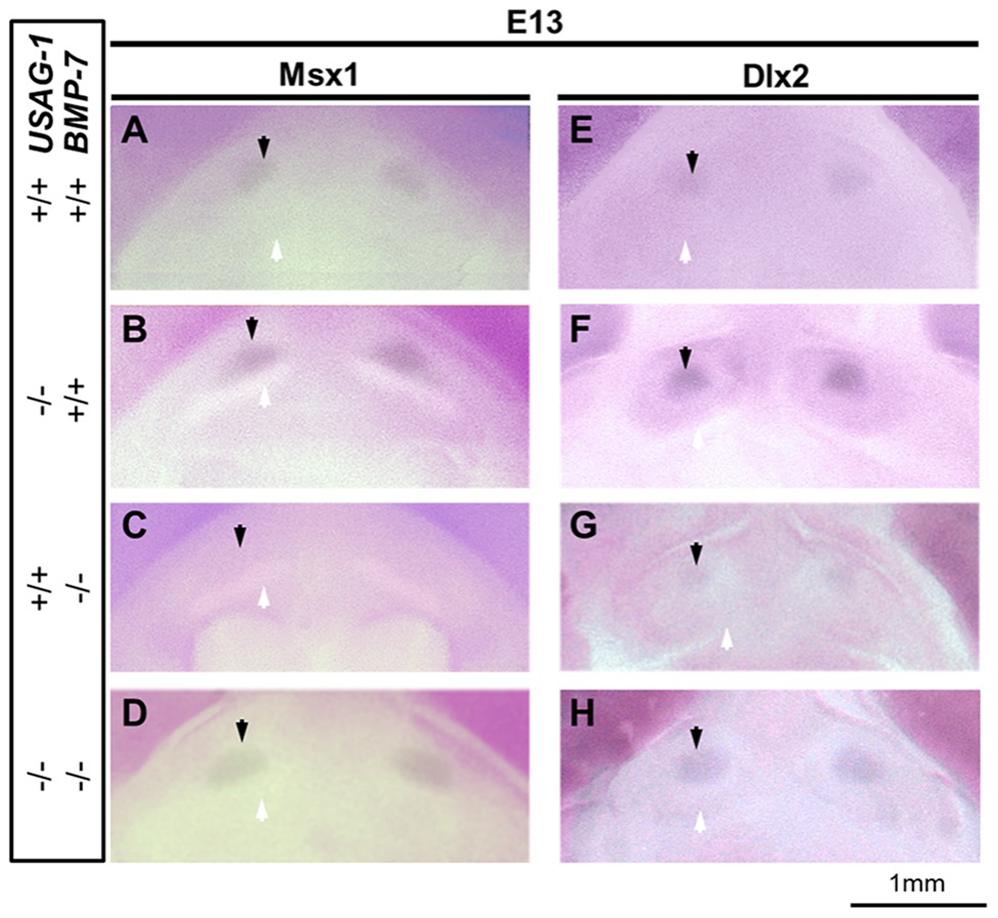
Intensive expression of Msx1 and Dlx2 in the rudimentary incisors of USAG-1 and BMP-7 mutants. Occlusal view of the tooth organ of rudimentary maxillary incisor primordium at E13 on whole mount in situ hybridization (A–H). Msx1 (A–D) and Dlx2 (E–H) transcription factors were expressed in the tooth organ of rudimentary maxillary incisor (black arrow) in addition to the tooth organ of characteristic incisor (white arrow). At E13, Msx1 and Dlx2 expression in the rudimentary maxillary incisors of USAG-1^−/−^/BMP-7^−/−^ mice was comparable with that of USAG-1^+/+^/BMP-7^+/+^, whereas that of USAG-1^−/−^/BMP-7^+/+^ appeared more intense as compared with that of controls (A–H).

**Figure 5:**
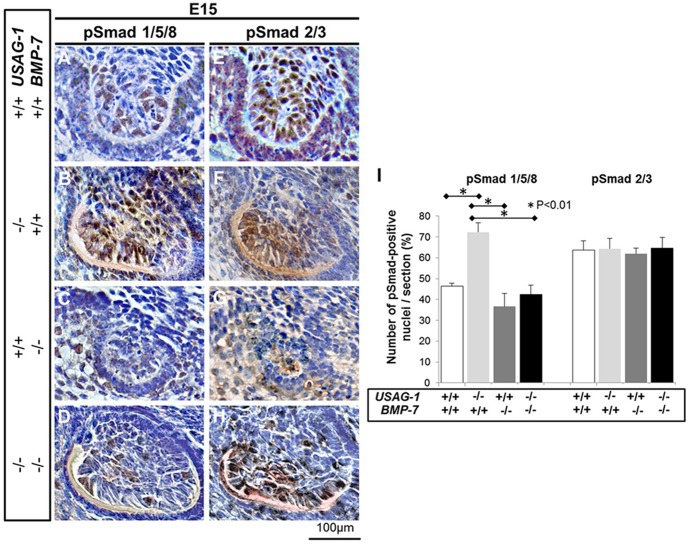
Enhanced BMP signal transduction in maxillary incisors of USAG-1 and BMP-7 mutants. Immunolocalisation of phosphorylated Smad (1/5/8 (A–D) and Smad 2/3 (E–H) at E15. USAG-1^+/+^/BMP-7^+/+^, (A, E); USAG-1^−/−^/BMP-7^+/+^, (B, F); USAG-1^+/+^/BMP-7^−/−^, (C, G) and USAG-1^−/−^/BMP-7^−/−^ (D, H). The number of pSmad 1/5/8– and pSmad 2/3- positive nuclei per section was counted in transverse sections of USAG-1^+/+^/BMP-7^+/+^ (white bars), USAG-1^−/−^/BMP-7^+/+^ (right grey bars), USAG-1^+/+^/BMP-7^−/−^ (dark grey bars), and USAG-1^−/−^/BMP-7^−/−^ (black bars) mice (n = 5; I). Compared with USAG-1^−/−^/BMP-7^+/+^ embryos, USAG-1^−/−^/BMP-7^−/−^ embryos iibited increased phosphorylated Smad 1/5/8- positive cells in odontogenic mesenchymal cells within the rudimentary maxillary incisor primordia at E15 (A–D). Employed immunostaining using anti-phospho-Smad 2/3 showed no difference among mutant mice (E–H). Enhanced BMP signalling in supernumerary teeth of the USAG-1-deficient mice could be inhibited by BMP-7 abrogation.

To evaluate whether increased BMP signaling in supernumerary teeth of the USAG-1 deficient mice could be prohibited by BMP-7 abrogation, we examined the Msx1 and Dlx2 expression; both of these transcription factors are downstream target genes of BMP-mediated signal transcription during tooth development at E13 [52], [53], with complementary phosphorylation of Smad 1/5/8 attributable to increased BMP signaling at E13 [21], [54] in USAG-1^+/+^/BMP-7^+/+^, USAG-1^−/−^/BMP-7^+/+^, USAG-1^+/+^/BMP-7^−/−^ and USAG-1^−/−^/BMP-7^−/−^ mice. At E13, Msx1 and Dlx2 expression in the rudimentary maxillary incisors of USAG-1^−/−^/BMP-7^−/−^ mice was comparable with that of USAG-1^+/+^/BMP-7^+/+^, whereas that of USAG-1^−/−^/BMP-7^+/+^ appeared more intense as compared with that in controls (Fig. 4A-H). Further, compared with USAG-1^−/−^/BMP-7^+/+^ embryos, USAG-1^−/−^/BMP-7^−/−^ embryos inhibited increased phosphorylated Smad 1/5/8 based upon positive odontogenic mesenchymal cells within the rudimentary maxillary incisor tooth primordia at E15 (Fig. 5A-D). To determine the specificity of phosphorylation of Smad 1/5/8, we employed immunostaining using anti-phospho-Smad 2/3, and found no difference among mutant mice (Fig. 5E-H). We conclude that increased BMP signaling in supernumerary teeth of the USAG-1 deficient mice is prohibited by BMP-7 abrogation.

### BMP-7 induces maxillary supernumerary incisors formation partially but not fully *in vitro*

**Figure 6:**
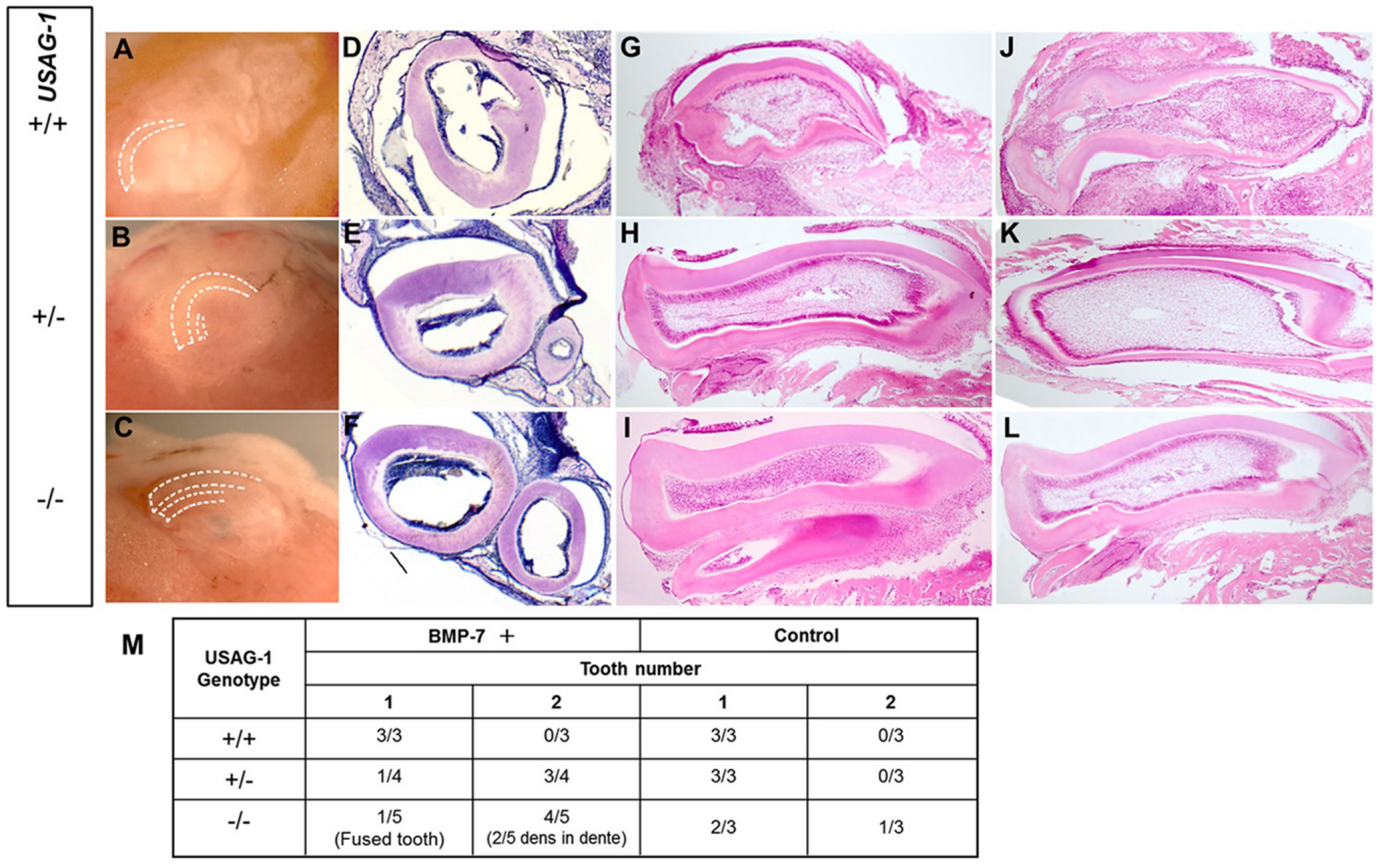
BMP-7 has potential to partially induce the formation of maxillary supernumerary incisors formation in vitro. Enhanced BMP-7 rescue the formation of maxillary incisor supernumerary tooth in E15 USAG-1 mutant mice in organ culture and subrenal capsule assay. The incisor explants supplemented with BMP-7 in USAG-1^+/−^ (E and H) and USAG-1^−/−^ (F and I) have supernumerary tooth in similar incidence after 20 days of culture, whereas these cultured explants in USAG-1^+/+^ (D and G) maintained the normal tooth number. (A–C) Explant appearance. (D–F) Coronal and (G–I) sagittal sections of explant. (J–L) Sagittal sections of control explant. (M) Table showing the relationship between number of teeth of explants and USAG-1 phenotypes.

To test whether BMP-7 actually induces supernumerary tooth formation, we performed explant culture and subsequent subrenal kidney capsule transplantation of E15 USAG-1 mutant maxillary incisor tooth primordia supplemented with BMP-7. We previously showed that the USAG-1^+/−^ mice showed phenotypically normal tooth number and position in maxillary incisor as well as wild type [15]. The incisor explants supplemented with BMP-7 in USAG-1^+/−^ as well as USAG-1^−/−^ have supernumerary tooth in similar numbers after 20 days culture, while these cultured explants in USAG-1^+/+^ presented normal tooth number (Fig.6A–J). These results demonstrated BMP-7 has a partial potential to induce supernumerary tooth formation, however it was not readily observed to induce extra tooth organs only with BMP-7.

## Discussion

Rudimentary organs are biological structures that appear to have no function as first described by Darwin in The Descent of Man [55]. Darwin listed so-called “wisdom teeth, the appendix, and the coccyx as rudimentary organs. Curiously, Reptiles with teeth as well as most mammals have complete dentitions with Rodentia (mice, rats, hamsters) and Lagomorphs (rabbits) which both present the unique diastema extending from incisor to molar tooth organs in the maxilla as well as mandible. Rather than the diastema truly representing a “toothless” region, a number of studies confirmed that the region in fact does contain rudimental primitive tooth organs at the bud stage of development [56]–[58]. Tooth organs, comparable to many other epidermal organs, are initiated as a placode and then progress through exquisite epithelial-mesenchymal interactions, reflecting a temporal and spatial sequence of unique signal transduction-mediated developmental processes [2], [6], [59]–[64].

In our present study, BMP-7 was co-localized with USAG-1 in the area of the maxillary rudiment incisor tooth germ in addition to the regular maxillary incisor tooth organ. USAG-1 abrogation rescued the apoptotic elimination of mesenchymal cells in the rudimentary maxillary incisor tooth primordia at E15, whereas the tooth sizes were comparable [15]. The apoptotic mesenchymal cells in USAG-1^−/−^/BMP-7^−/−^are similar to USAG-1^+/+^/BMP-7^+/+^ in contrast to that of USAG-1^−/−^/BMP-7^+/+^. These results support our interpretation that USAG-1 functions as a novel BMP-7 antagonist in the maxilla. We confirmed that increased BMP signaling in supernumerary teeth of the USAG-1 deficient mice could be prohibited by BMP-7 abrogation. In the contrast, to test whether BMP-7 has the potential to induce supernumerary tooth formation, we performed explant culture and subsequent subrenal kidney capsule culture. The incisor explants supplemented with BMP-7 in USAG-1^+/−^ as well as USAG-1^−/−^ have supernumerary tooth in similar numbers after 20 days culture, while these cultured explants in USAG-1^+/+^ retained normal tooth number. These results demonstrated that BMP-7 can induce supernumerary tooth formation, however it is impossible to induce extra tooth by only BMP-7. Finally, we conclude that gene interactions between BMP-7 and USAG-1 regulate the supernumerary maxillary incisor formation.

The supernumerary incisors documented in mutant mice have been located on the lingual side of the normal incisor [15], [17], [23], [65], or side-by-side [8], [66]–[69]. The Spry2^+/−^/Spry4^−/−^ mice indicated two separate incisors in two different enamel organs located side by side, in which supernumerary incisor development was shown in vivo to result from the second splitting of the incisor primodium [69]. The duplicated incisors belong to the same generation. Within these supernumerary incisor formation side-by-side, the β-cat^△Prx/lacZ^ mice also present two incisors that each belong to the same generation, but in these mice only the lower incisor have been reported to be affected [68]. The mechanisms of supernumerary formation appear to be different between maxilla and mandibular morphogenesis.

A detailed analysis of USAG-1 deficient mice showed that the supernumerary incisor developed on the lingual side of the normal one, and this tooth was considered to belong to a different tooth generation [15], [23]. The supernumerary incisor of Lrp4 deficient mice have the same origin as the supernumerary incisor of USAG-1 mutants [17]. We previously demonstrated that the supernumerary maxillary incisor was the result of the survival and successive development of the rudimentary incisor tooth primordia, and that USAG-1 controls the number of teeth in the maxillary incisor region by regulating apoptotic elimination of odontogenic mesenchymal cells [15].

Further, it was reported that the supernumerary mandibular incisor corresponded to the revival of the replacement incisor by regulating apoptosis of odontogenic epithelial cells [23]. These results suggest that the potential mechanism by which supernumerary incisor on the lingual side of the normal incisor is different between maxilla and mandible. In USAG-1 single deficient mouse, supernumerary teeth were observed in 100% of the maxillary incisor regions, whereas partial penetrance was observed in the mandible. We demonstrated that USAG-1 acted as BMP-7 antagonist in supernumerary maxillary incisor formation, and absence of the maxillary teeth of conditional BMP-7 null mice [44]. The expression of USAG-1 and BMP-7 is opposite around the rudimentary incisor tooth primordia between maxilla and mandible (Fig. S1). In addition, in mature adult mice, supernumerary teeth can be induced on both labial and lingual sides of the incisors, regions which contain adult stem cells supporting the continuous growth of mouse incisors [22], [70]. In young mice, supernumerary tooth organs were induced in multiple regions adjacent to both incisor and molar regions. Presumably, supernumerary tooth organs can form directly from the oral epithelium, in the dental lamina connecting the developing molar or incisor tooth organs to the oral epithelium, in the crown region, and even in the elongating and furcation area of the developing root [22].

In the rudimentary maxillary incisor of BMP-7 deficient mouse, specific phenotypic alterations are found. In approximately half of the embryos studied, the rudimentary maxillary incisors were discovered to be missing. Defects in odontogenesis have been reported in several mouse mutants for genes associated with BMP as well as other signaling pathways [3], [71]. Deletion of Alk3 (BMPR1a) in the epithelium leads to tooth development arrest at the bud stage [72], indicating the importance of mesenchyme-derived BMP signals for the further development of the dental epithelium. The epithelial overexpression of Noggin, which is an antagonist of the BMP signaling, results in various phenotypic alterations including lack of mandibular molars, reduced number of maxillary molars, disrupted root size and pattern, as well as poorly mineralized enamel [73]. In Msx1-deficient mice tooth development is arrested at the cup stage [74], a phenotype that can be rescued by administration of BMP-4 [75]. In vitro, BMP-4 and BMP-7 can both induce the expression of Msx1 and Msx2 as shown by the implantation of BMP-releasing beads into the mouse molar mesenchyme [52], [76]. The present report provides the direct functional evidence of a nonredundant role for BMPs in tooth initiation and development. The fact that the observed phenotypes are not fully penetrant could be explained by a partial redundancy where other BMPs or other signaling molecules compensate for BMP-7. As BMPs show different affinities for the various type I BMP receptors, a molecular discrimination between signals initiated by different BMPs under physiological conditions is expected. An indication of the importance of BMP-7 for aspects as variable as tooth induction, patterning, and development comes from observations showing different degree of phenotype penetrance in incisors vs. molars as well as in maxillary teeth vs. mandibular teeth. The molecular networks that determine rodent tooth specification (i.e. molars and incisors, maxillary and mandibular teeth) involve genes such as the Islet1, Pitx1, Barx1, and Dlx [77]–[79], thus integrating BMP-7 into their pathway.

The presence of epithelial anlagen of the third dentition was also noticed in human [80]–[82]. The epithelium which is considered as the anlagen of the third dentition develops lingual to all permanent tooth germs [83]. Furthermore, when it appears, the predecessor (permanent tooth germ) is in the bell-shaped stage [83]. The time of appearance of the third dentition seems after birth. This means that we have chance to access the formation of the third dentition in the mouth. Recently, a number of mouse mutant are now starting to provide some insights into the mechanisms of supernumerary tooth formation. Multiple supernumerary teeth may have genetic components in their etiology and represent partial of the third dentition in humans. Such candidate molecules or genes might be those that are involved in embryonic tooth induction, in successional tooth formation or in the control of the number of the teeth [2].

The supernumerary tooth formation using genetically-defined mouse models clearly demonstrate the feasibility to induce de novo tooth formation by in situ repression or activation of a single candidate gene. Our investigations and related support or validate the hypothesis that de novo repression or activation of candidate genes such as BMP-7 or USAG-1 could be used to stimulate a third dentition to induce or achieve new tooth regeneration in mammals. In vivo gene delivery could be the suitable gene therapy approach in the tooth regeneration by stimulation of a third dentition.

## Conclusions

The mechanism for suppressing deciduous incisors in mice is expression of USAG-1, which inhibits BMP-7 signaling, leading to apoptosis and degeneration of rudimentary tooth germs. The dental phenotypes of USAG-1 and BMP-7 mutants reported by our studies provide a rationale for future tooth regeneration.

## Supporting Information

Figure S1 The expression of USAG-1 and BMP-7 in the lower jaws. USAG-1 and BMP-7 expression in mandibular incisor primordia. (A–D) Whole-mount X-Gal expression in tooth germs of E14 and E15 mandibular. (A’–D’) Parasagittal sections (anterior to the left) of the tooth germs. USAG-1 (A–B, A’–B’) and BMP-7 (C–D, C’–D’) were expressed in the tooth organ of rudimentary mandibular incisor (red arrow) in addition to the tooth organ of characteristic incisor (black arrow). At E14, USAG-1 started to be expressed in the epithelial cells of the mandibular rudimentary and regular incisor primordia (A and A’). At E15, the expression of USAG-1 continued in the epithelium (B and B’). In the meantime, the expression of BMP-7 localized mesenchymal cells of mandibular rudimentary and regular incisor primordia at both E14 and E15 (C, D, C’ and D’).
(TIF)

Table S1 Summary of tooth phenotype.
(DOCX)
