# Supplementary figures and images for: Interactions between BMP-7 and USAG-1 (Uterine Sensitization-Associated Gene-1) Regulate Supernumerary Organ Formations
Source: PLoS One. 2014 May 9;9(5):e96938. doi: 10.1371/journal.pone.0096938 (PMC4016158; doi:10.1371/journal.pone.0096938)

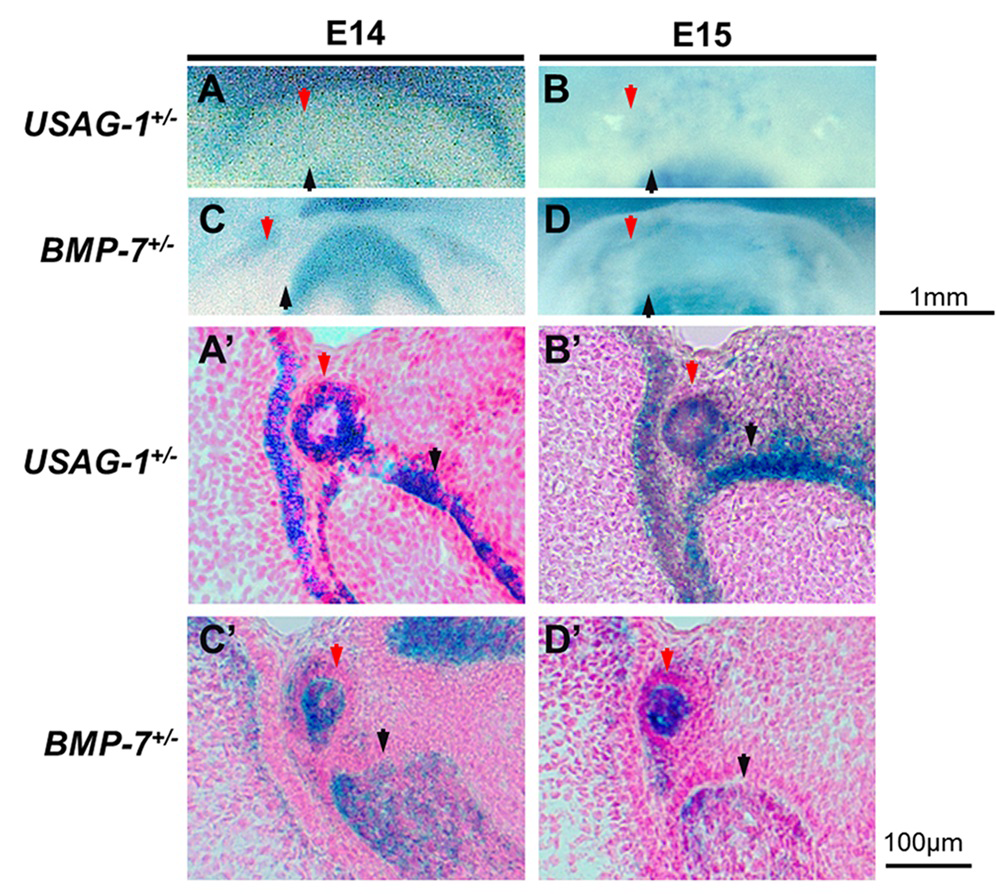

Supplement: Figure S1 — The expression of USAG-1 and BMP-7 in the lower jaws. USAG-1 and BMP-7 expression in mandibular incisor primordia. (A–D) Whole-mount X-Gal expression in tooth germs of E14 and E15 mandibular. (A’–D’) Parasagittal sections (anterior to the left) of the tooth germs. USAG-1 (A–B, A’–B’) and BMP-7 (C–D, C’–D’) were expressed in the tooth organ of rudimentary mandibular incisor (red arrow) in addition to the tooth organ of characteristic incisor (black arrow). At E14, USAG-1 started to be expressed in the epithelial cells of the mandibular rudimentary and regular incisor primordia (A and A’). At E15, the expression of USAG-1 continued in the epithelium (B and B’). In the meantime, the expression of BMP-7 localized mesenchymal cells of mandibular rudimentary and regular incisor primordia at both E14 and E15 (C, D, C’ and D’). (TIF) [file pone.0096938.s001.tif]

Supplemental Table S1

Summary of tooth phenotype


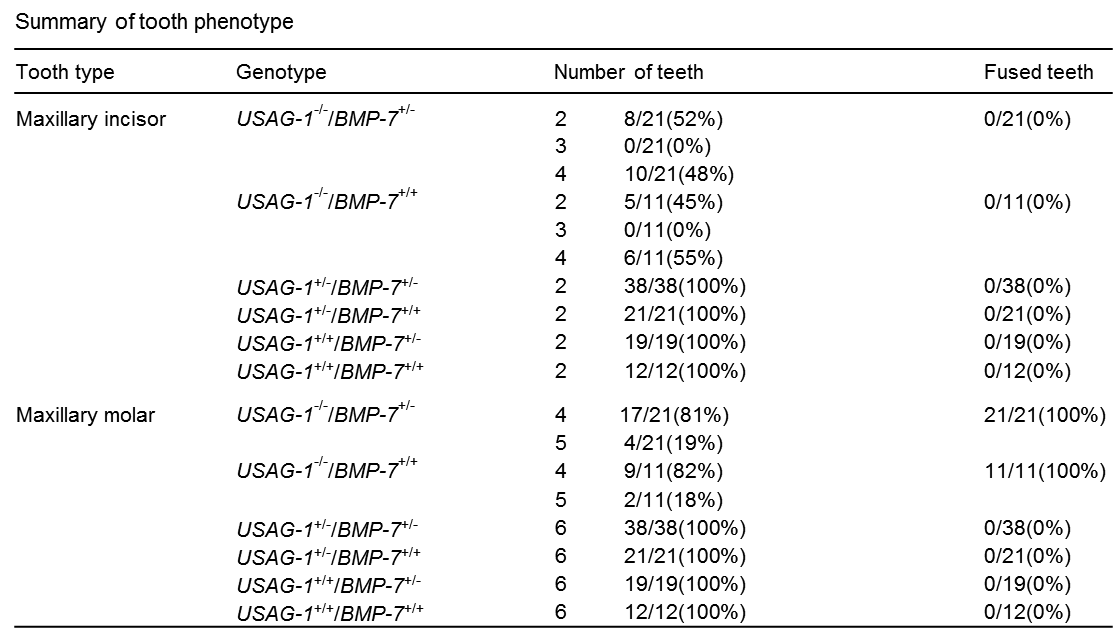

Supplement: Table S1 — Summary of tooth phenotype. (DOCX) [file pone.0096938.s002.docx]
